# Evolutionary regime transitions in structured populations

**DOI:** 10.1371/journal.pone.0200670

**Published:** 2018-11-26

**Authors:** Fernando Alcalde Cuesta, Pablo González Sequeiros, Álvaro Lozano Rojo

**Affiliations:** 1 Instituto de Matemáticas, Universidade de Santiago de Compostela, E-15782 Santiago de Compostela, Spain; 2 Departamento de Didácticas Aplicadas, Facultade de Formación do Profesorado, Universidade de Santiago de Compostela, Avda. Ramón Ferreiro s/n, E-27002 Lugo, Spain; 3 Centro Universitario de la Defensa, Academia General Militar, Ctra. Huesca s/n, E-50090 Zaragoza, Spain; 4 IUMA, Universidad de Zaragoza, Pedro Cerbuna 12, E-50009 Zaragoza, Spain; 5 GeoDynApp - ECSING Group, E-15782 Santiago de Compostela, Spain; Queen Mary University of London, UNITED KINGDOM

## Abstract

The evolutionary dynamics of a finite population where resident individuals are replaced by mutant ones depends on its spatial structure. Usually, the population adopts the form of an undirected graph where the place occupied by each individual is represented by a vertex and it is bidirectionally linked to the places that can be occupied by its offspring. There are undirected graph structures that act as *amplifiers of selection* increasing the probability that the offspring of an advantageous mutant spreads through the graph reaching any vertex. But there also are undirected graph structures acting as *suppressors of selection* where this probability is less than that of the same individual placed in a homogeneous population. Here, firstly, we present the distribution of these evolutionary regimes for all undirected graphs with *N* ≤ 10 vertices. Some of them exhibit transitions between different regimes when the mutant fitness increases. In particular, as it has been already observed for small-order random graphs, we show that most graphs of order *N* ≤ 10 are amplifiers of selection. Secondly, we describe examples of amplifiers of order 7 that become suppressors from some critical value. In fact, for graphs of order *N* ≤ 7, we apply computer-aided techniques to symbolically compute their fixation probability and then their evolutionary regime, as well as the critical values for which they change their regime. Thirdly, the same technique is applied to some families of highly symmetrical graphs as a mean to explore methods of suppressing selection. The existence of suppression mechanisms that reverse an amplification regime when fitness increases could have a great interest in biology and network science. Finally, the analysis of all graphs from order 8 to order 10 reveals a complex and rich evolutionary dynamics, with multiple transitions between different regimes, which have not been examined in detail until now.

## Introduction

In recent times the evolutionary theory on graphs has become a key field to understand biological systems. Although evolutionary dynamics has been classically studied for homogeneous populations, there is now a wide interest in the evolution of populations arranged on graphs after mutant spread. The process transforming vertices occupied by residents into vertices occupied by mutants is described by the *Moran model*. Introduced by Moran [1] as the Markov chain that counts the number of invading mutants in a homogeneous population, it was adapted to subdivided population by Maruyama [2, 3] and rediscovered by Lieberman et al. [4] for general graphs. For undirected graphs where edges have no orientation, mutants will either become extinct or take over the whole population, reaching one of the two absorbing states, *extinction* or *fixation*. The *fixation probability* is the fundamental quantity in the stochastic evolutionary analysis of a finite population.

If the population is homogeneous, at the beginning, one single vertex is chosen at random to be occupied by a mutant individual among a population of *N* resident individuals. Afterwards, an individual is randomly chosen for reproduction, with probability proportional to its reproductive advantage (1 for residents and *r* ≥ 1 for mutants), and its clonal offspring replaces another individual chosen at random. In this case, the fixation probability is given by

$$\begin{array}{c} \Phi_{0}\left(r\right)=\frac{1-r^{-1}}{1-r^{-N}}=\frac{r^{N-1}}{r^{N-1}+r^{N-2}+\cdots +r+1}. \end{array}$$ (1)

If the population is arranged on vertices of an undirected graph, the replacements are limited to the vertices that are connected by edges. According to the Isothermal Theorem [2–4], the fixation probability Φ(*r*) = Φ_0_(*r*) if and only if the graph is *isothermal* (i.e. the temperature *T*_*i*_ = ∑_*j*∼*i*_ 1/*d*_*j*_ of any vertex *i* is constant, where *j* is a neighbor of *i* and *d*_*j*_ is the number of neighbors of *j*), or equivalently *regular* (i.e. the degree *d*_*i*_ of any vertex *i* is constant). But there are graph structures altering substantially the fixation chances of mutant individuals depending on their fitness. As showed in [4], there are graph structures that *amplify* this advantage. This means the fixation probability function Φ(*r*) > Φ_0_(*r*) for all *r* > 1 for the same order *N*. Notice that Φ(1) = 1/*N* and the inequality must be reversed for *r* < 1. Due to the exact analytical computation of the probability Φ(*r*) given by Monk et al. [5], it is known that star and complete bipartite graphs are *amplifiers of natural selection* whose fixation functions are bounded from above by

$$\begin{array}{c} \Phi_{2}\left(r\right)=\Phi_{0}\left(r^{2}\right)=\frac{1-r^{-2}}{1-r^{-2N}}. \end{array}$$ (2)

**Fig 1:**
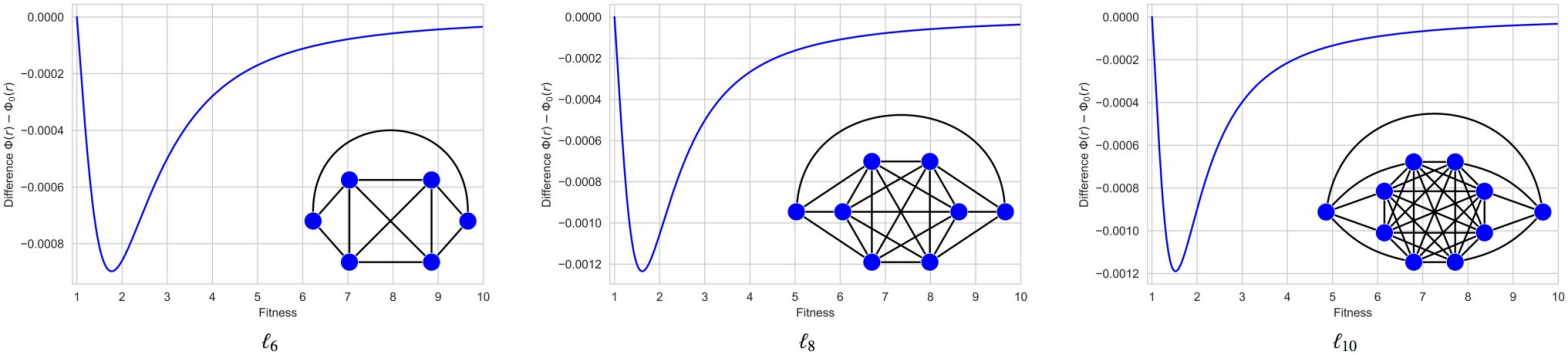
Suppressors of order 6, 8 and 10 for any fitness value. In [7], we called *ℓ-graph* the undirected graph of even order *N* = 2*n* + 2 ≥ 6 obtained from the clique *K*_2*n*_ by dividing its vertex set into two halves with *n* ≥ 2 vertices and adding 2 extra vertices. Each of them is connected to one of the halves of *K*_2*n*_ and with the other extra vertex. The *ℓ*-graphs *ℓ*_6_, *ℓ*_8_, and *ℓ*_10_ are shown in the figure, together with the functions Φ(*r*) − Φ_0_(*r*) which have been symbolically computed to evidence the suppression of selection.

On the other hand, there are also graph structures that *suppress* the reproductive advantage of mutant individuals so that Φ(*r*) < Φ_0_(*r*) for all *r* > 1. Examples of this kind of graph structures were known for some fitness values (see [6]). In [7], we presented examples of *suppressors of natural selection* of order 6, 8 and 10, denoted by *ℓ*_6_, *ℓ*_8_ and *ℓ*_10_, whose fixation probabilities remain smaller than Φ_0_(*r*) for every *r* > 1 (see Fig 1).

**Fig 2:**
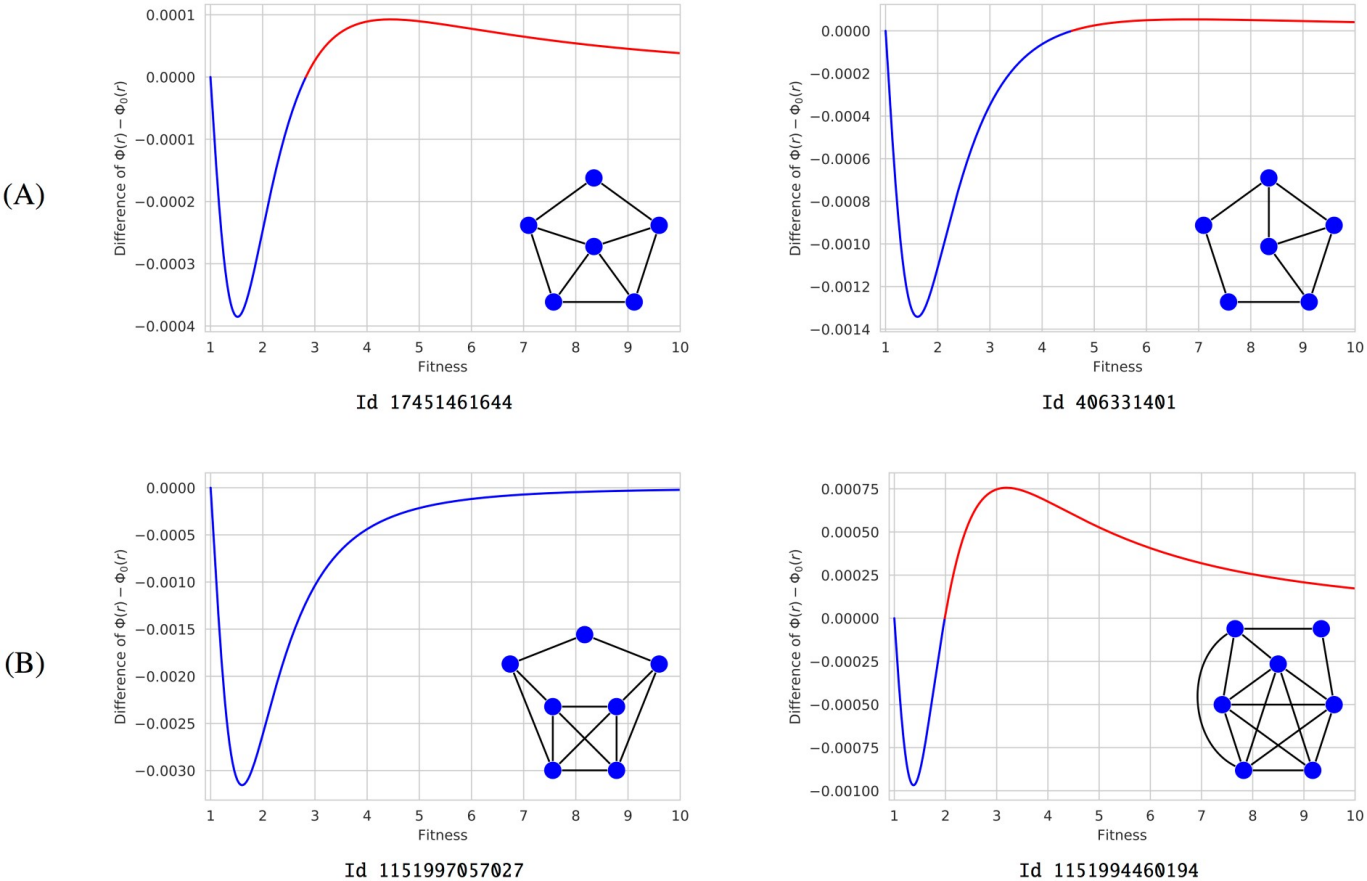
Suppressors that become amplifiers. (A) Examples of order 6 from [11] with a unique transition at *r*_*c*_ ≈ 2.82 and *r*_*c*_ ≈ 4.56 respectively. (B) Examples of order 7 from [6] having a unique transition at *r*_*c*_ ≈ 79.15 and *r*_*c*_ ≈ 1.98 respectively. In both cases, we use identification numbers from [11] to facilitate any search in our database. Each graph is shown with the function Φ(*r*) − Φ_0_(*r*), which has been symbolically computed.

The analysis of disadvantageous mutants (with *r* < 1) is also interesting when comparing amplification and suppression of selection for graphs, but here, for simplicity, we focus on the case of advantageous mutant (with *r* > 1). On the other hand, different initialization and updating types have been also considered in [8] and [9], see also [10] for a comparative analysis of update mechanisms. If the initial distribution is uniform (i.e. the probability that a vertex will be occupied by the initial mutant is equal for all the vertices) and the graph evolves under Birth-Death updating, Hindersin and Traulsen showed in [9] that almost all small undirected graphs are amplifiers of selection. Assuming both conditions and focusing on the advantageous case, we distinguish two different evolutionary regimes (out of the isothermal one): given values 1 ≤ *r*_0_ < *r*_1_ ≤ +∞, a graph is an *amplifier of selection for r* ∈ (*r*_0_, *r*_1_) if the fixation probability function Φ(*r*) > Φ_0_(*r*) and a *suppressor of selection for r* ∈ (*r*_0_, *r*_1_) if Φ(*r*) < Φ_0_(*r*) for all *r*_0_ < *r* < *r*_1_. Star and bipartite complete graphs are amplifiers for *r* ∈ (1, +∞) and graphs *ℓ*_6_, *ℓ*_8_ and *ℓ*_10_ are suppressors for *r* ∈ (1, +∞). There are also suppressors that become amplifiers from some critical value *r*_*c*_ > 1 (see Fig 2). In this case, we say *r*_*c*_ is a *transition* between both evolutionary regimes. In general, we say that the evolutionary dynamics of a structured population presents a *transition* at *r*_*c*_ > 1 if there are values 1 ≤ *r*_0_ < *r*_1_ ≤ +∞ such that the graph is a suppressor (resp. an amplifier) for *r* ∈ (*r*_0_, *r*_*c*_) and an amplifier (resp. a suppressor) for *r* ∈ (*r*_*c*_, *r*_1_).

In this paper, we show the complete distribution of the three evolutionary regimes (isothermal, amplifier and suppressor) for all graphs of order 10 or less, exactly for order 7 or less and extremely accurate for fitness values varying from 0.25 to 10 with step size of 0.25 for other orders. In particular, we corroborate a previous observation by Hindersin and Traulsen [9] on random graphs of small order by showing that most graphs of order 10 or less are amplifiers or suppressors that become amplifiers from a unique transition *r*_*c*_ > 1.

The exhaustive identification of suppressors in order 6 and 7 allows us to describe a suppression mechanism similar to that of *ℓ*-graphs [7] and *clique-wheels* [12]. The existence of topological configurations favoring the suppression of selection opens the way to the search of specific suppression mechanisms in biological networks.

We also exhibit two other types of transitions which might also have important consequences:

- There are amplifiers of order greater or equal to 7 that become suppressors from a unique transition *r*_*c*_ > 1.
- There are graphs of order greater or equal to 8 exhibiting more than one transition.

For finite populations, it has been suggested that results obtained for weak selection may remain valid when the selection is no longer weak. However, in [13], Wu et al. showed that this is no the case for homogeneous populations under frequency dependent selection. Here, we show that the phenomenon can happen in a structured population even when the selection is frequency independent. Moreover, the fact that the survival chances of mutant individuals may decrease with respect to a homogeneous population when their fitness increases might have interesting biological consequences.

In fact, a family of graphs of order ≥ 12 that change from amplifier into suppressor as *r* increases has recently been shown in [14] by using numerical simulation. But, for the three graphs of order 7 that exhibit this change of regime, we know that there is a unique transition as we have symbolically computed their fixation probability for any *r* > 1. We focus on two graphs which are constructed from the same building blocks and we analyze some possible generalizations by computing the fixation probability, either symbolically when they have enough symmetries or solving the system of linear equations (with extreme precision for a large range of fitness values) otherwise.

Because of the expected properties of the rational functions Φ_0_(*r*) and Φ(*r*) (as monotonically increasing functions with decreasing derivatives), the last two results were not exactly expected. But the existence of multiple crosses for these curves leads us to consider these properties that will be commented in the discussion section (see also S1 Text). The non-uniqueness of transitions also implies that numerical simulation is not enough to determine the evolutionary regime of a graph. Indeed, if there are no transitions or if there is only one transition, we can infer the regime of a graph of the simulation for a narrow range of fitness values. But if there are graphs with multiple changes of regime, it is not possible.

## Materials and methods

In [11], we presented an extremely precise database of fixation probabilities for all undirected graphs of order 10 or less for fitness values *r* varying from 0.25 to 10 with step size of 0.25 (see details below). From the analysis of this database, we firstly detected suppressors described in [7], and later regime transitions described here. Initially, we identified two amplifiers of order 7 with a transition to the suppression regime at some *r*_*c*_ ≤ 10. Then we envisaged to describe the complete distribution of the different evolutionary regimes (isothermal, amplifier, and suppressor) of all graphs of order 10 or less using the barcode technique. This has revealed that undirected graphs have a complex and rich evolutionary dynamics that are worth studying in detail. We have also adapted the technique described in [7] (implementing it in C++, see S1 File) to symbolically compute the fixation probability Φ(*r*) of all graphs of order 7 or less determining their evolutionary regime for any fitness value *r* > 1 (see also details below). Thus, we have found a third example of order 7 with a transition from amplifier to suppressor at some *r*_*c*_ > 10, and we have proved that these three examples really have a unique transition. The same method has been also applied to some graphs of greater order generalizing suppressors and amplifiers that change into suppressors in order to detect some possible suppression mechanisms. When they have not enough symmetries to symbolically compute their fixation probability, we have proceed according to [11], but solving the system of linear equations for additional fitness values from 10 to 2,000 with step size of 1).

### Mathematical model

Let *G* be an undirected graph with vertex set *V* = {1, …, *N*}. In fact, all graphs considered here will be assumed connected. Denote by *d*_*i*_ the degree of the vertex *i*. The *Moran process* on *G* is a Markov chain *X*_*n*_ whose states are the vertex sets *S* inhabited by mutant individuals at each time step *n*. The transition probabilities are obtained from the matrix *W* = (*w*_*ij*_) given by *w*_*ij*_ = 1/*d*_*i*_ if *i* and *j* are neighbors and *w*_*ij*_ = 0 otherwise. More precisely, for each fitness value *r* > 0, the transition probability between *S* and *S*′ is given by

$$\begin{array}{c} P_{S,S^{\prime }}\left(r\right)=\{\begin{array}{cc} \frac{r\sum_{i\in S}w_{ij}}{w_{S}\left(r\right)} & \text{if}\;S^{\prime }\backslash S=\left\{j\right\},\\ \frac{\sum_{i\in V\backslash S}w_{ij}}{w_{S}\left(r\right)} & \text{if}\;S\backslash S^{\prime }=\left\{j\right\},\\ \frac{r\sum_{i,j\in S}w_{ij}+\sum_{i,j\in V\backslash S}w_{ij}}{w_{S}\left(r\right)} & \text{if}\;S=S^{\prime },\\ 0 & \text{otherwise}, \end{array} \end{array}$$ (3)

where

$$\begin{array}{c} w_{S}\left(r\right)=r\sum_{i\in S}\sum_{j\in V}w_{ij}+\sum_{i\in V\backslash S}\sum_{j\in V}w_{ij}=r\left|S\right|+N-\left|S\right| \end{array}$$ (4)

is the total reproductive weight of resident and mutant individuals. The *fixation probability* of each subset *S* ⊂ *V* inhabited by mutant individuals

$$\begin{array}{c} \Phi_{S}\left(r\right)=P\left[\;\exists n\geq 0:X_{n}=V|X_{0}=S\;\right] \end{array}$$ (5)

is a solution of the system of 2^*N*^ linear equations

$$\begin{array}{c} \Phi_{S}\left(r\right)=\sum_{S^{\prime }}P_{S,S^{\prime }}\Phi_{S^{\prime }}\left(r\right) \end{array}$$ (6)

with boundary conditions Φ_∅_(*r*) = 0 and Φ_*V*_(*r*) = 1. The *(average) fixation probability* is given by

$$\begin{array}{c} \Phi \left(r\right)=\frac{1}{N}\sum_{i=1}^{N}\Phi_{\left\{i\right\}}\left(r\right). \end{array}$$ (7)

Denoting by ***P***(*r*) = (*P*_*S*,*S*′_(*r*)) the transition matrix, Eq 6 can be written as

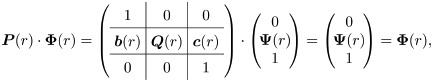
(8)
with respect to the block decomposition of ***P***(*r*) and **Φ**(*r*) obtained from the decomposition of the power set P(V) into absorbing states *S* = ∅, *V* and non-absorbing states *S* ≠ ∅, *V*. In other words, **Φ**(*r*) = (0, **Ψ**(*r*), 1) is the vector with coordinates Φ_*S*_(*r*), (1, ***b***(*r*), 0) is the vector with coordinates *P*_*S*,∅_(*r*), and (0, ***c***(*r*), 1) is the vector with coordinates *P*_*S*,*V*_(*r*) for any subset *S* ⊂ *V*. It can be also rewritten as

$$\begin{array}{c} (I-Q(r))\cdot \Psi (r)=c(r), \end{array}$$ (9)

where ***I*** is the identity matrix of size 2^*N*^ − 2. This equation has a unique solution **Ψ**(*r*) whose coordinates are rational functions on *r* with rational coefficients [7, S1 Text]. But considering Eq 3, we can multiply the equation associated to each state *S* by *w*_*S*_(*r*) reducing Eq 9 to

$$\begin{array}{c} Q^{*}\left(r\right)\cdot \Psi \left(r\right)=c^{*}\left(r\right), \end{array}$$ (10)

where the entries of Q*(*r*) and c*(*r*) are now degree one polynomials with rational coefficients.

### Database

In [11], we presented an accurate database of the fixation probabilities for all connected undirected graphs with 10 or less vertices, which means 11,989,763 graphs excluding the trivial one with one single vertex. The generation of the edge lists was done with SageMath, whereas the computation of Φ(*r*) was written in the C programming language. Firstly, we compute the matrix Q*(*r*) and the vector c*(*r*). Since their entries are polynomials of degree one with rational and positive coefficients, they can be represented as two pairs of 64 bits integers. Therefore there is no precision loss in this step. Next, we evaluate Q*(*r*) and c*(*r*) for each fitness value *r* varying from 0.25 to 10 with step size of 0.25, and solve Eq 10 with a high relative precision LU decomposition algorithm (relative errors for isothermal and complete bipartite graphs, used as benchmarks, are less that 10^14^, see [11] for details). Each graph is identified (up to isomorphism) with a unique 64 bits unsigned integer, which allows us to recover the edge list without previous knowledge of its order or size, see again [11] and references therein for details. The database is available from [15].

### Computation method

A method to compute the exact (average) fixation probability Φ(*r*) of graphs of small order with symmetries was described in [7]. As we already said, Φ(*r*) = Φ′(*r*)/Φ″(*r*) is a rational function where the numerator $\Phi^{\prime }(r)=\sum_{i=0}^{d}a_{i}r^{i}$ and the denominator $\Phi^{\prime \prime }(r)=\sum_{i=0}^{d}b_{i}r^{i}$ are polynomials with rational coefficients of degree *d* ≤ 2^*N*^ − 2. Symmetries are used to bound the degree *d* and therefore the number 2(*d* + 1) of coefficients involved in the computation of Φ(*r*). Since Φ(*r*) converges to 1 as *r* → +∞, we can assume *a*_*d*_ = *b*_*d*_ = 1 and then Eq 6 can be replace with a system of 2*d* linear equations

$$\begin{array}{c} \sum_{i=0}^{d}a_{i}r^{i}=\Phi \left(r\right)(\sum_{i=0}^{d}b_{i}r^{i}) \end{array}$$ (11)

that arise from evaluating Φ(*r*) for fitness values *r* ∈ {1, …, *d* + 1, 1/2, …, 1/*d*}. There is some sort of indetermination on the system due to the fact that Φ′(*r*) and Φ″(*r*) may have common factors. Then, one could pick up any solution of the system (they are different representations of the same rational function) or reduce the bound of *d* until one have one single solution (corresponding to the canonical representation of the rational function with coprime numerator and denominator). We developed a C++ program with this algorithm, available from S1 File, which allows us to symbolically

- compute the fixation probability Φ(*r*) for these values, and
- solve the reduced linear system Eq 11.

Once Φ(*r*) has been computed solving this system, we can determine the regimes and the transitions of the graph by computing the sign and zeros of the rational function Φ(*r*) − Φ_0_(*r*).

## Results

As explained by Hindersin and Traulsen in [9], the early constructed examples of amplifiers and suppressors seem suggest that it could be easier to construct suppressors of selection than amplifiers of selection. It is true when one focuses on directed graphs, but as shown in [9], most undirected (Erdös-Rényi) random graphs of small order are amplifiers of selection under Birth-Death updating. Here, we corroborate this observation by showing that most undirected graphs of order *N* ≤ 10 are amplifiers of selection for fitness values *r* ≤ 10. Furthermore, we describe the distribution of isothermal graphs, amplifiers of selection, and suppressors of selection for fitness values varying from 0.25 to 10 with step size of 0.25. In fact, for the 996 graphs of order 7 or less, the fixation probability has been symbolically computed (see Computation Method). Results are gathered in Table 1, which is one of our main results. The number, type and place of transitions for all graphs of order 7 or less are given in S1 Table. We can observe that transitions do not only occur at high values of the fitness, but also at values close to *r* = 1. On the other hand, to confirm the number of transitions in greater orders, we have enlarge the range of fitness values (varying now from 10 to 2,000 with step size of 1) for which the system of linear equations Eq 6 is solved. Even so, as we have specified in the introduction, the number of amplifiers and suppressors of selection is only apparent since the fitness values are limited to a more or less large interval.

**Table 1:** Number and percentage of isothermal and suppressor graphs, as well as graphs exhibiting one or more transitions. Graphs are determined up to isomorphism, so any graph cannot be mapped to each other via a permutation of vertices and edges. For graphs of order 6 and 7, the fixation probability has been symbolically computed to exactly give type and place of each transition. These data are gathered in S1 Table. All exact results are marked in bold. However, for graphs of order 8 and more, we must distinguished between suppressors and ‘apparent suppressors’ as fitness values only vary between 1 and 10. Despite this, additional computations for some higher values of the fitness *r* (varying from 10 to 2,000 with step size of 1) seem exclude more than two transitions in order 8 and 9 and more than three transitions in order 10.

N	#	Iso	Sup	1 Trans	2 Trans	3 Trans
**6**	**112**	**5 (4.46%)**	**1 (0.89%)**	**6 S/A (5.36%)**		
**7**	**853**	**4 (0.47%)**	**3 (0.35%)**	**52 S/A (6.10%)** **3 A/S (0.35%)**		
**8**	**11,117**	**17 (0.15%)**	90 (0.81%)	427 S/A (3.84%) 36 A/S (0.32%)	3 S/A/S (0.03%)	
**9**	**261,080**	**11 (<0.01%)**	1,951 (0.75%)	9,489 S/A (3.63%) 854 A/S (0.33%)	43 S/A/S (0.02%) 6 A/S/A (<0.01%)	
**10**	**11,716,571**	**167 (<0.01%)**	91,110 (0.78%)	407,001 S/A (3.47%) 40,974 A/S (0.35%)	3,086 S/A/S (0.03%) 578 A/S/A (<0.01%)	19 S/A/S/A (<0.01%)

**N** = Order, **#** = Number, **Isotherm** = Isothermal, **Sup** = Suppressor, **Trans** = Transition, **S/A** = Transition from suppressor to amplifier, **A/S** = Transition from amplifier to suppressor, **S/A/S** = Double transition Suppressor/Amplifier/Suppressor, **A/S/A** = Double transition Amplifier/Suppressor/Amplifier, **S/A/S/A** = Triple transition Suppressor/Amplifier/Suppressor/Amplifier.

Thus, in order 6, there are exactly one suppressor of selection, namely the graph *ℓ*_6_ described in [7] (see Fig 1), five isothermal graphs, and six suppressors that become amplifiers from a unique critical value. The remaining 100 graphs are amplifiers of selection. Two of suppressors changing into amplifiers was already described in [11] (see Fig 2(A)). All the graphs portrayed in the paper are gathered in S1 and S2 Figs with indication of their identification numbers, names, regimes and transitions.

### Amplifiers and suppressors of order 7

**Fig 3:**
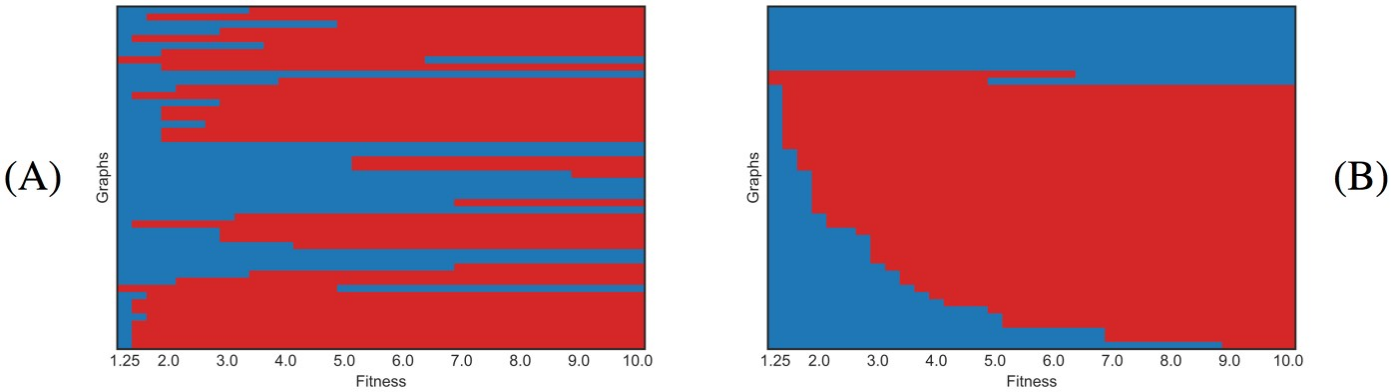
Barcodes describing regime transition of graphs of order 7. Each horizontal line corresponds to a graph, and color represents the evolutionary regime for the given fitness: blue color corresponds to the suppression regime and red color to the amplification regime. (A) Unsorted data for suppressors and graphs with one transition. (B) Sorted data for suppressors and graphs with one transition.

A close look to the barcode diagram for the 853 graphs of order 7 (as shown in Fig 3) reveals a new phenomenon: we distinguish two amplifiers that become suppressors from a critical value *r*_*c*_ ≤ 10. From the symbolic computation of the fixation probability Φ(*r*), we find three suppressors of selection, namely Id 1134281908237, Id 1134281902105, and Id 1151998128135, and a number of suppressors that later become amplifiers of selection, namely 52, including the suppressors presented in [6] and depicted in Fig 2(B). Moreover, we find indeed three amplifiers Id 1151592835082, Id 1151860745228, and Id 1151592837126 becoming suppressors at *r*_*c*_ ≈ 4.98, *r*_*c*_ ≈ 6.37 and *r*_*c*_ ≈ 24.79 respectively. A quantitative resume is given in Table 1.

Is the suppression of selection a specific property of each of graphs listed above? Or can one infer some suppression mechanism that could even reverse an amplification regime? To answer these questions, at least partially, we have focused on some of these graphs showing certain similarities.

### Regime transitions for *ℓ*-graphs

**Fig 4:**
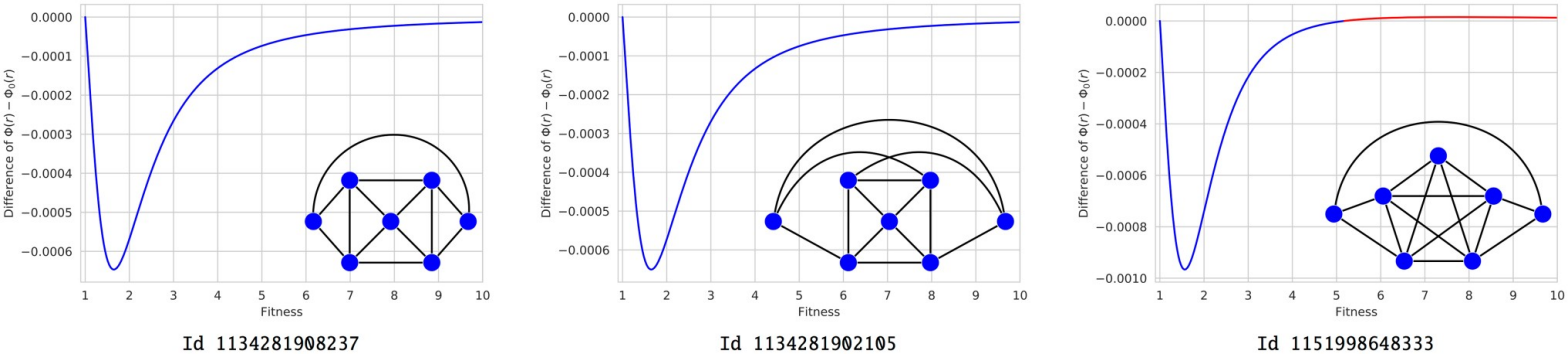
Suppressors for weak selection and beyond. From the symbolic computation of the differences Φ(*r*) − Φ_0_(*r*), we know that the graphs Id 1134281908237 and Id 1134281902105 are suppressors for any fitness value, while Id 1151998648333 exhibit a unique transition Suppressor/Amplifier at *r*_*c*_ ≈ 5.17.

The two first suppressors Id 1134281908237 and Id 1134281902105 are shown in Fig 4. For reasons of convenience, we have added a third graph Id 1151998648333 with a unique transition from the suppression to the amplification regime at *r*_*c*_ ≈ 5.17. Their construction is very similar to *ℓ-graphs* defined in [7]. Recall that $\ell_{N}=\ell_{N}^{n,n}$ is an undirected graph of even order *N* = 2*n* + 2 ≥ 6 obtained from the clique *K*_2*n*_ by dividing its vertex set into two halves with *n* ≥ 2 vertices and adding 2 extra vertices. Each of them is connected to one of the halves of *K*_2*n*_ and with the other extra vertex (see Fig 1). More generally, we denote by $\ell_{N}^{n,m}$ the undirected graph obtained adding two interconnected extra vertices to the clique *K*_*N*−2_ and connecting each one to disjoint families of vertices in the clique having *n* and *m* elements with *n* + *m* ≤ *N* − 2. We say that $\ell_{N}^{n,m}$ is *balanced* if *n* = *m* and *unbalanced* otherwise. As is also established in S1 Fig, the third graph depicted in Fig 4 is precisely $\ell_{7}^{2,2}$. Notice also that only the case *n* + *m* = *N* − 2 was considered in [7].

For the three graphs in Fig 4, the suppression mechanism seems directly related to the suppressor nature of *ℓ*-graphs [7] and *clique-wheels* [12]. Indeed, on a star graph, it is more likely that a peripheral mutant survives and reproduces occupying the central vertex. On the contrary, on a complete graph, it is much more unlikely that the initial mutant will survive surrounded by residents. But in *ℓ*-graphs and clique-wheels, the survival chances of the mutants placed at the central clique are reduced by its connection with peripheral vertices occupied by residents, as well those of the peripheral mutants connected with central residents, although a subtle balance seems to be needed to suppress the reproductive advantage of mutant individuals. To confirm this idea, we explore evolutionary regimes and transitions of some balanced and unbalanced *ℓ*-graphs.

In [7], we saw that a subtle balance in the peripheral connections was necessary to the global suppression. More precisely, some unbalanced *ℓ*-graphs of order 7, namely $\ell_{7}^{1,4}$ and $\ell_{7}^{2,3}$, were studied in [7] using Monte Carlo simulation. Both are suppressors: the first one changes into amplifier from a relatively small fitness value, whereas the second one remains a suppressor for any fitness value *r* ≤ 10. Now, due to the new symbolic computation, we know that both present a unique transition (from the suppression to the amplification regime) at *r*_*c*_ ≈ 1.80 and *r*_*c*_ ≈ 25.47 respectively. Therefore, we can surmise that only the balanced *ℓ*-graphs with *n* + *m* = 2*n* = *N* − 2 are global suppressors.

**Fig 5:**
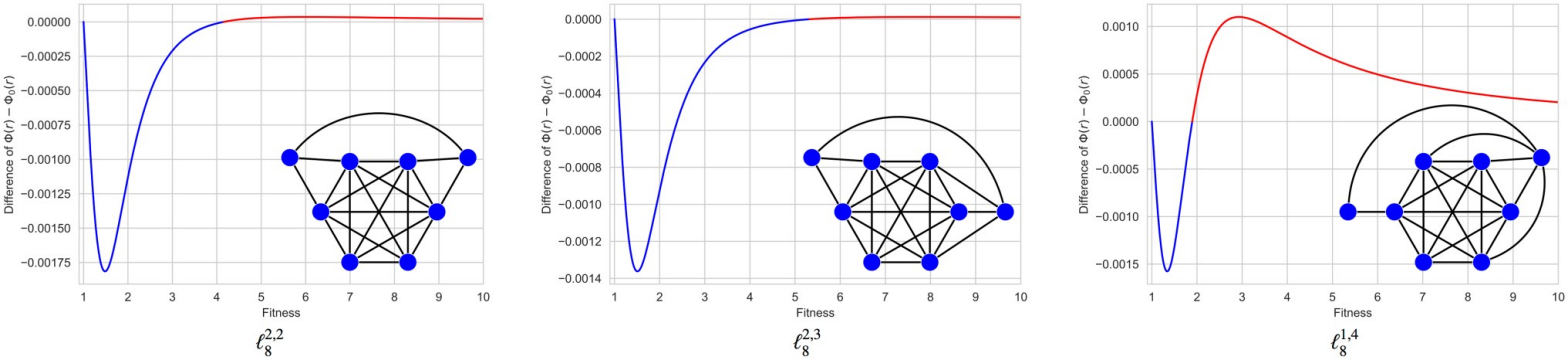
*ℓ*-graphs of order 8. From the symbolic computation of the differences Φ(*r*) − Φ_0_(*r*), we know that the graphs $\ell_{8}^{2,2}$, $\ell_{8}^{2,3}$ and $\ell_{8}^{1,4}$ (with identification numbers Id 38605195624473, Id 38605195632653 and Id 38605187250242) have a unique transition of type Suppressor/Amplifier at *r*_*c*_ ≈ 4.15, *r*_*c*_ ≈ 5.32 and *r*_*c*_ ≈ 1.89 respectively.

In order 8, we consider the graphs $\ell_{8}^{2,2}$, $\ell_{8}^{2,3}$, and $\ell_{8}^{1,4}$ which are represented in Fig 5. They still are suppressors that become amplifiers from critical values *r*_*c*_ ≈ 4.15, *r*_*c*_ ≈ 5.32 and *r*_*c*_ ≈ 1.89 respectively.

**Fig 6:**
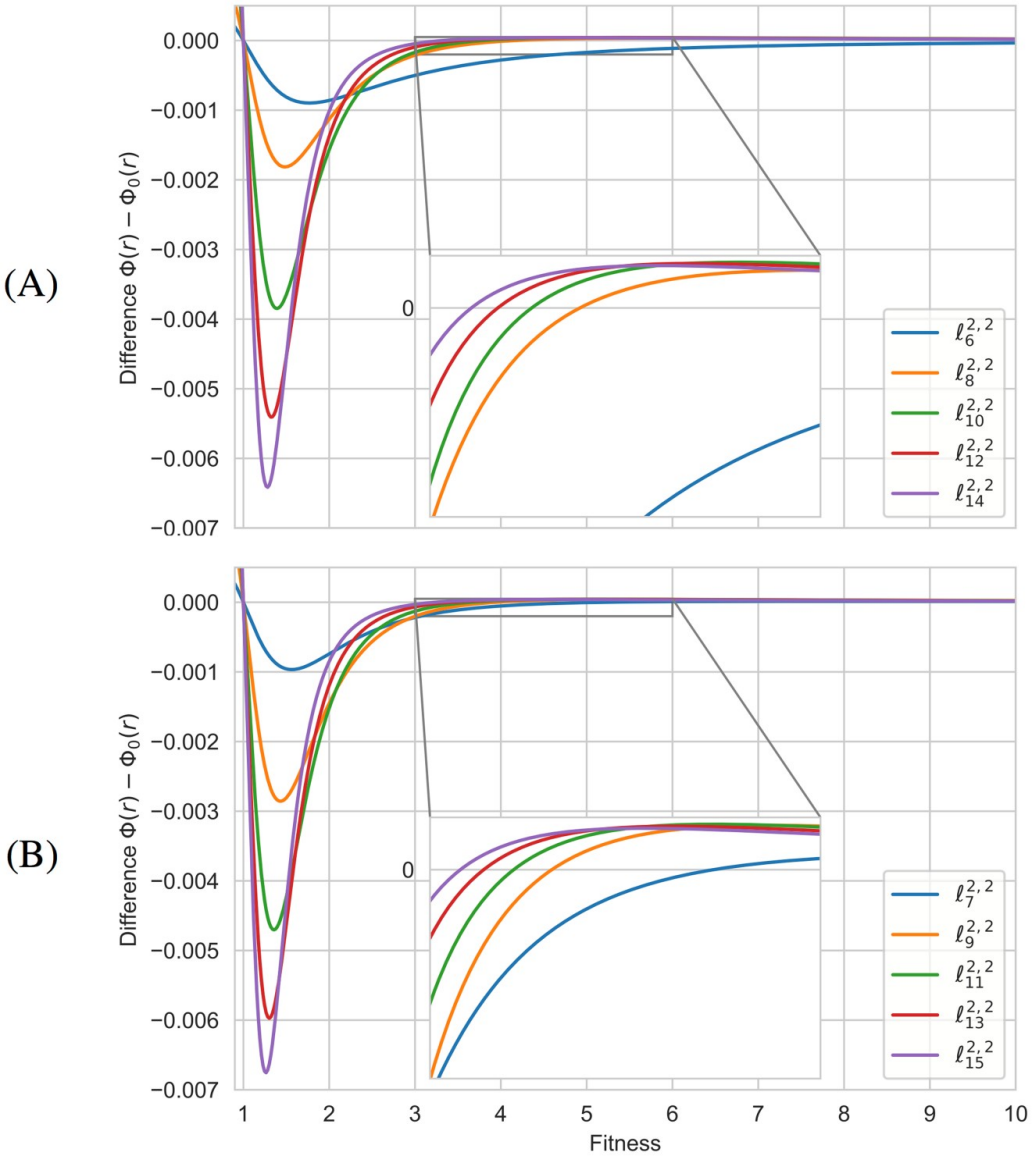
The exact differences Φ(*r*) − Φ_0_(*r*) for the graphs $\ell_{N}^{2,2}$. **(A) Even orders**. **(B) Odd orders**. Initial evolutionary regimes are distinguishable from the graphs of the functions Φ(*r*) − Φ_0_(*r*) associated to the graphs $\ell_{N}^{2,2}$ when *N* varies from 6 to 15, while transitions can be observed in the zoomed images. The exact places of transitions are specified in S2 Table.

Due to the symmetries, the symbolic computation is also applicable to the graphs $\ell_{N}^{2,2}$ when *N* varies from 6 to 15. The exact differences Φ(*r*) − Φ_0_(*r*) are depicted in Fig 6 although the monotonous behavior of transitions can be better seen in S2 Table. As before, all these graphs are suppressors with a unique transition to the amplification regime.

In summary, there are reasons to accept the existence of a suppression mechanism shared by *ℓ*-graphs and clique-wheels (in the sense of [12] and [14]), although we think that new techniques of potential theory on directed graphs will be probably required to identify any mathematical underlying principle. For this purpose, *ℓ*-graphs have some interest since their state spaces (described explicitly in [7]) are simpler than those of the family of clique-wheels. We ignore if this particular mechanism could has biological interest (although somewhat similar rules have been detected in neural networks), but we think that the existence of suppression mechanisms (especially those that reverse the amplification of selection when the fitness increases) has a real interest in biology and network science.

### Transitions from the amplification to the suppression regime

**Fig 7:**
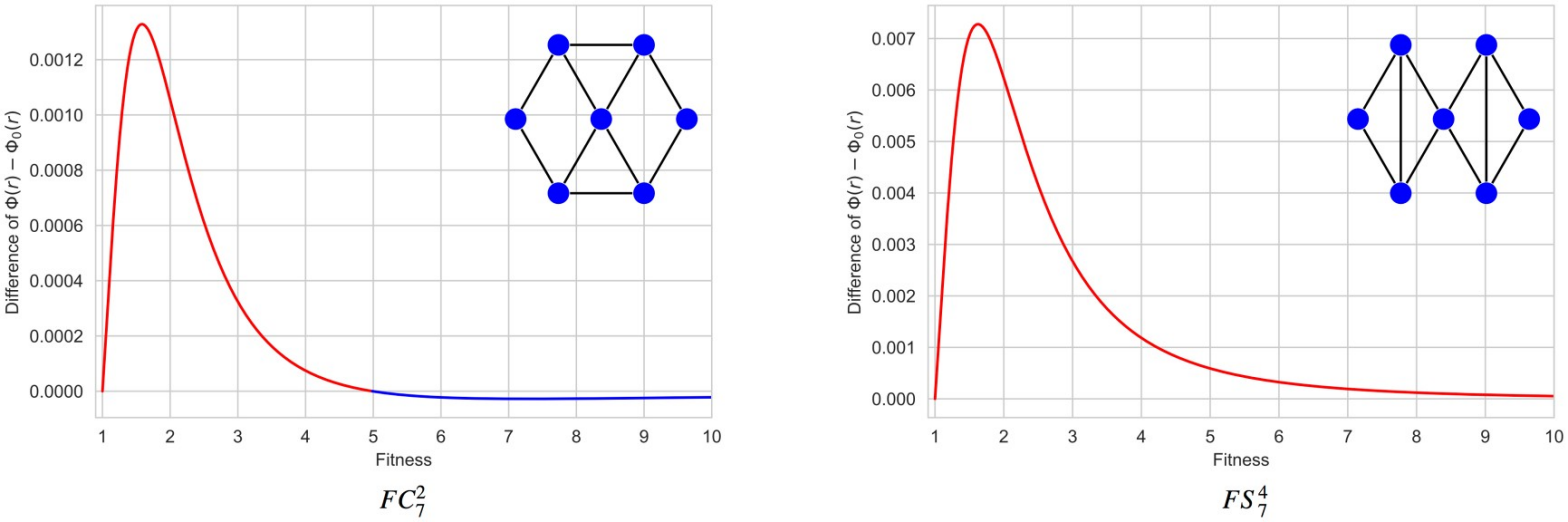
Amplifiers that become suppressors. From the symbolic computation of the differences Φ(*r*) − Φ_0_(*r*), we know that the friendship cycle $FC_{7}^{2}$ (with identification number Id 1151592835082) becomes a suppressor from *r*_*c*_ ≈ 4.98, and the friendship star $FS_{7}^{4}$ (with identification number Id 1151592837126) becomes a suppressor from *r*_*c*_ ≈ 24.79.

As we have already said, in order 7, there are three amplifiers Id 1151592835082, Id 1151860745228, and Id 1151592837126 that become suppressors from critical values *r*_*c*_ ≈ 4.98, *r*_*c*_ ≈ 6.37 and *r*_*c*_ ≈ 24.79. According to our aim, we have focused on the first and the last of these graphs which are constructed from the same building blocks (see Fig 7). As before, we would like to infer some suppression mechanism from the analysis of these specific examples.

**Fig 8:**
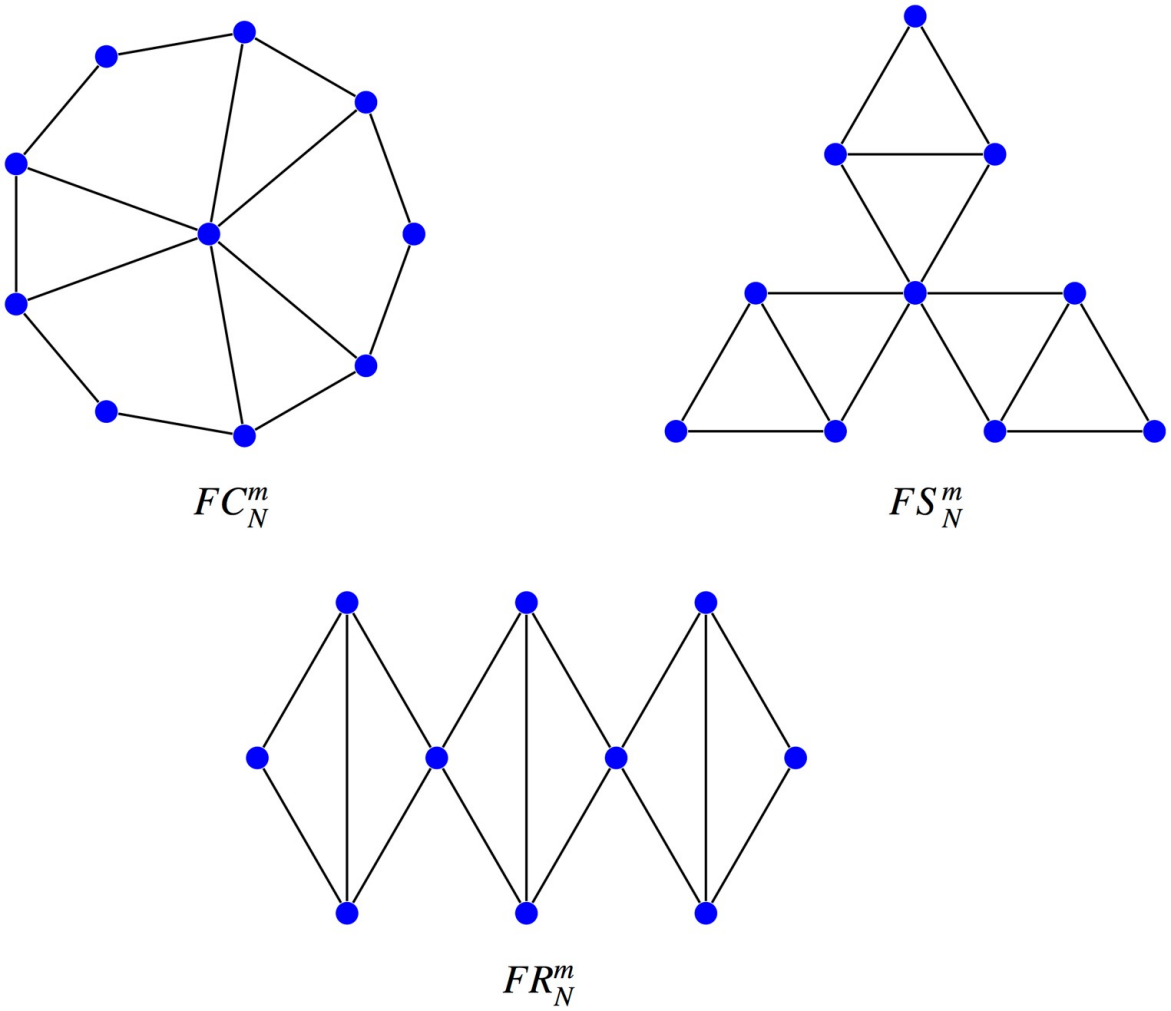
Friendship cycles, stars, and ribbons. The friendship cycle $FC_{N}^{m}$, the friendship star $FS_{N}^{m}$ and the friendship ribbon $FR_{N}^{m}$ where *N* is the number of vertices and *m* is the number of 3-cliques.

We say that Id 1151592835082 is a *friendship cycle* $FC_{7}^{2}$ and Id 1151592837126 is a *friendship star $FS_{7}^{4}$*. A friendship cycle $FC_{N}^{m}$ is a cycle *C*_*n*_ where *m* disjoint pairs of neighbors are connected to an extra central vertex so that two vertices have at most one neighbor in common, the total order *N* being equal to *n* + 1. In a friendship star $FS_{N}^{m}$, two neighbors can share two different neighbors, but they cannot be connected either to each other, nor to another new neighbor. Additionally, one single vertex can belong to more than two 3-cycles composing a friendship subgraph (to distinguish it from a friendship ribbon $FR_{N}^{m}$ where no vertex can belong to more than two 3-cliques, see Fig 8). Here *N* is the number of vertices and *m* is the number of 3-cliques.

**Fig 9:**
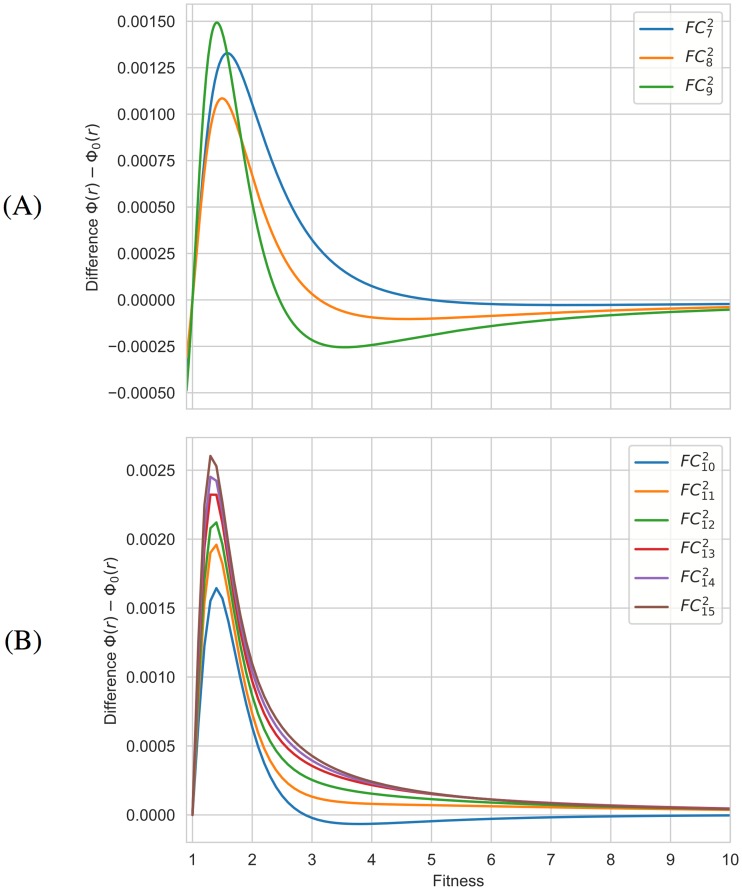
The differences Φ(*r*) − Φ_0_(*r*) for the friendship cycles $FC_{N}^{2}$ with *N* varying from 7 to 15. (A) For *N* = 7, 8, 9, the differences Φ(*r*) − Φ_0_(*r*) have been symbolically computed and therefore they are exact. (B) For 10 ≤ *N* ≤ 15, the differences Φ(*r*) − Φ_0_(*r*) have been computed by reducing the number of linear equations and solving the reduced system for the usual fitness values between *r* = 1 and *r* = 10. They are not exact, but extremely accurate.

We have symbolically computed the fixation probability Φ(*r*) for the friendship cycles $FC_{N}^{2}$ with *N* = 7, 8, 9 proving that they are amplifiers transformed into suppressors from fitness values *r*_*c*_ ≈ 4.98, *r*_*c*_ ≈ 3.12, and *r*_*c*_ ≈ 2.45 respectively (see Fig 9(A)). For greater orders *N* varying from 10 to 15, we have used the symmetries to reduce the size of the system of linear equations Eq 6 and then we have solved the system for the usual fitness values (from 0.25 to 10 with steps of 0.25). The differences Φ(*r*) − Φ_0_(*r*) are shown in Fig 9(B). The graph $FC_{10}^{2}$ shows a transition in the interval [1, 10], but it is not clear that the others friendship cycles $FC_{N}^{2}$ evolve from the amplification to the suppression regime. Similarly, the friendship cycle $FC_{10}^{3}$ (see S2 Fig) is an amplifier that becomes a suppressor from *r*_*c*_ ≈ 4.09.

On the other hand, we have also symbolically computed Φ(*r*) for the friendship star $FS_{10}^{6}$ showing that it becomes a suppressor at *r*_*c*_ ≈ 9.96. However, since there are not enough symmetries to reduce the system of equations, this approach could not be pushed any further. As this also happens to $FR_{10}^{6}$, we have computed the fixation probability by solving the system of linear equation Eq 6 for the usual fitness values, seeing that $FR_{10}^{6}$ remains an amplifier for any *r* ≤ 10. Then we have extended the interval to *r* = 2000 without finding any transition, so this is probably a global amplifier. Both graphs are also depicted in S2 Fig.

As before *ℓ*-graphs, friendship cycles and friendship stars seem to illustrate new mechanisms of suppression, whose underlying principles need to be studied and compared with those of *clique-arms* described in [14].

### Amplifiers and suppressors of order 8 and more

Another interesting phenomena is also revealed by the barcode diagrams in order greater or equal to 8: there are multiple transitions as shown in S3, S4 and S5 Figs. The existence of double transitions of type Suppressor/Amplifier/Suppressor is clearly visible in S3 Fig. The three graphs of order 8 with double transition are portrayed in S6 Fig. For graphs of order 9, to this type of double transitions we must add a few transitions of type Amplifier/Suppressor/Amplifier, as shown in S4 Fig. A number of double transitions (of both types) is also shown in S5 Fig for the graphs of order 10. Note the emergence of regular patterns that suggest some kind of regularity in the distribution of transitions. But in fact, as shown in S5 Fig, there are triple transitions for a few graphs of order 10. As we said before, the number of transitions are only apparent because we cannot be sure that there will not exist other transitions for these orders. However, extending the fitness values for which the system of linear equation Eq 6 is solved, we have confirmed that there is no new transitions between *r* = 10 and *r* = 2000 for these few graphs. Subject to this caveat, Table 1 gives a detailed account of the proportion of amplifiers and suppressors of selection, as well as that of graphs exhibiting one or more transitions.

## Discussion

Initially motivated by our interest in the robustness of biological and technological networks against invasion [16], we decided to compute the fixation probability of all graphs with 10 vertices or less, totaling 11,989,764 graphs, to facilitate general searches without specific aims. As explained in Materials and Methods, we solved (by Gaussian elimination) the system of linear equations Eq 6 for each graph and for each fitness value varying from 0.25 to 10 with step size of 0.25. Collected data has been published in [15] and details has been explained in [11].

Our first search focused on extremal graphs (from the point of view of the evolutionary regime), and in this way we found some graph structures suppressing the advantage of mutant individuals occupying their vertices for any fitness value. This property seems particularly appealing for biological networks like brain and protein-protein interaction networks, but also in the tumor initiation process within healthy tissues as proposed in [17]. Most graph structures reduce in a very slight amount the advantage of a invading mutant, but some suppression mechanisms could be amplified by repetitive rules (such as those described in [18] and [19] for neuronal networks) involved in the modular architecture of many biological networks.

More concretely, in [7], we have developed symbolical and numerical computations to show the suppressing nature of a family of graphs, called *ℓ-graphs*, which generalizes the only suppressor *ℓ*_6_ of order 6.

From the serendipitous discover of amplifiers that change into suppressors, we proposed ourselves to identify the global distribution of amplifiers and suppressors of selection, with or without transitions, for order 10 or less. We have used the database mentioned above for this purpose, showing in particular that most graphs of order 10 or less are amplifiers of selection. In spite of the complexity of some amplifier structures [4] and the effort required to prove that nature [5, 6, 8, 20, 21], this confirms the observation made by Hindersin and Traulsen [9] for random graphs of small order.

Some suppressors that become amplifiers were also known, but we exhibit here new examples (gathered in S1 Fig) whose suppression mechanism seems similar to that of clique-wheels [12] and *ℓ*-graphs [7]. However, for graphs of order *N* = 7, there is another type of transition: we initially found two amplifiers that become suppressors from critical values *r*_*c*_ ≈ 4.98 and *r*_*c*_ ≈ 6.37. But for greater orders, the change of evolutionary regime is more amazing because some graphs exhibit more than one transition. As before, for *N* = 8, these graphs has been detected from the barcodes diagram S3 Fig and then identified and represented in S6 Fig by means of the database [15]. All double transitions are identical of type Suppressor/Amplifier/Suppressor.

Contrary to the idea that results obtained for weak selection may remain valid out of this case (see [13] and references therein for a discussion about this problem), these observations indicate that some graph structures can dramatically alter the evolutionary regime of a structured population, even reversing the amplification of the survival likelihood of advantageous mutants, as their fitness increases. In our opinion, this fact has important biological and theoretical implications.

As the analytical computation of the fixation probability for these graphs does not seem feasible for now, we have adapted the method described in [7] running a new C++ program (see S1 File) to symbolically compute the fixation probabilities of all graphs of order 7 or less. This method has been also applied to compute the fixation probability of some graphs of greater order with enough symmetries. In particular, this has allowed us to exactly determine the number and the place of transitions for these orders, gathered in Table 1 and S1 Table, which constitute one of the main results of the paper. In this way, we have also found a third amplifier of order 7 with a transition at *r*_*c*_ ≈ 24.79, and consequently we have become interested in the evolutionary regime of friendship cycles and friendship stars portrayed in Fig 8.

More generally, for graphs of order *N* = 9, only simple and double transitions are visible in S4 Fig, whereas triple transitions are distinguishable in S5 Fig for order *N* = 10. In the first case, there are 6 graphs with double transitions of type Amplifier/Suppressor/Amplifier among a total of 49 graphs with more than one transition. In the second one, we found 19 graphs with triple transitions, all of the same type Suppressor/Amplifier/Suppressor/Amplifier. All these remarks are part of Table 1. Given the number of graphs of order 9 and 10 having some double or triple transition, it would be tedious (and hard without enough symmetries) to symbolically determine the exact number of transitions for each graph, so it cannot be excluded the (unlikely) existence of new transitions. However, additional computations for higher fitness values (varying from 10 to 2,000 with step size of 1) seem exclude this possibility.

As we already said, although transitions between different evolutionary regimes were known (see [6, 9, 11] and [14]), theses results reveal that undirected graphs have a complex and rich evolutionary dynamics admitting multiple transitions between different regimes. This poses new challenges in computing fixation probabilities and times because the numerical simulation cannot always provide accurate answers to extremal problems on fixation probabilities, nor probably on absorption or fixation times. In other words, from the simulation on a narrow range of fitness values, we cannot infer the persistence of a certain evolutionary regime for higher values. Only analytical or symbolical computation can evidence the persistence of such regime.

Our techniques can be easily adapted to compute absorption or fixation times for graphs of small order with enough symmetries. Analyzing the case of disadvantageous mutants (with *r* < 1) is feasible with the same techniques. Although we have focused our analysis on the average fixation probability, all computations have been made separately once the initial placement of the mutant has been fixed. How this placement affect the evolutionary regime depending on the fitness is something that can be described, whether all vertices have the same probability to be chosen or this probability is proportional to the temperature. The last initialization procedure, called *temperature initialization* in [8], is perhaps is more plausible from a biological point of view.

More difficult, however, is to change the updating method from Birth-Death to Death-Birth because, while the fixation probabilities are still rational functions, the involved polynomials are in general of higher degree. The underlying reason is that one cannot multiply by a reproductive weight to obtain a system of equations as in Eq 10. This makes the computation more difficult or even unfeasible.

Finally, all barcode diagrams, but specially those of graphs of order 10, show very particular patterns in the distribution of transitions, which are worth exploring. On the other hand, the existence of multiple transitions has been established, but this fact is somewhat surprising. Initially, we thought that the functions Φ_0_(*r*) and Φ(*r*) were not only increasing, but also concave functions for *r* > 1 (i.e. with negative second derivative for all *r* > 1), so we did not expect multiple crossings. Now we know that Φ_0_(*r*) is not concave for all population sizes, but only for *N* = 2, 3, 4, 5.

In the limit for large populations and for weak selection, we have uniform convergence in class *C*^0^, but not in class *C*^1^. So qualitative changes may appear near *r* = 1. Furthermore, from the symbolic computation of first and second derivative of Φ(*r*) for any graph of order *N* ≤ 7, we know that all functions associated to graphs or order 4 or less are concave. In order 5, the complete graph and the cycle (the other graph of constant degree) are concave. In fact, *r* = 1 is an inflection point. For degree greater than 5, the map Φ_0_(*r*) is convex around *r* = 1 and becomes concave at some point (see S1 Text).

So, what are the features of the graphs leading to multiple transitions and their particular distribution is an important issue for future work. But the main challenge is to translate differential features of suppressors (as it has been achieved with some isothermal graphs and amplifiers) into features of their state spaces in order to compute their fixation probabilities and to identify the mathematical principles on which their suppression mechanisms are based.

## Supporting information

S1 Text On some properties of Φ_0_(*r*) and its limit as *N* → ∞. Proofs of some facts on the concavity of the function Φ_0_(*r*).
(PDF)

S1 File C++ program. To compute the fixation probabilities of small order graphs for any fitness value *r* > 1, we ran a new C++ program available from https://bitbucket.org/geodynapp/phasetransition.
(ZIP)

S2 File Exact fixation probabilities of all graphs of order 7 or less. This repository contains the output of S1 File, and information about the first and second derivative of all studied graphs, including all graphs of 7 or less vertices. Available from https://bitbucket.org/geodynapp/fixationfunctions.
(ZIP)

S1 Table Regime transitions from order 2 to order 7. Number, place, and type of transitions for all connected graphs of order 7 or less.
(PDF)

S2 TableRegime transitions for graphs $\ell_{N}^{2,2}$ from order 6 to order 15.(PDF)

S1 Fig Suppressors for weak selection (and beyond). All the figures representing suppressors are gathered with indication of their identification numbers, names, regimes and transitions.
(EPS)

S2 FigFriendship cycles, ribbons and stars.Some friendship cycles $FC_{N}^{m}$, stars $FS_{N}^{m}$, and ribbons $FR_{N}^{m}$ of order *N* = 7 and 10 are compared.(EPS)

S3 Fig Barcodes describing regime transitions of graphs of order 8. Each horizontal line corresponds to a graph, and color represents the evolutionary regime for the given fitness: blue color corresponds to the suppression regime and red color to amplification regime.
(EPS)

S4 Fig Barcodes describing regime transitions of graphs of order 9. Each horizontal line corresponds to a graph, and color represents the evolutionary regime for the given fitness: blue color corresponds to the suppression regime and red color to amplification regime.
(EPS)

S5 Fig Barcodes describing regime transitions of graphs of order 10. Each horizontal line corresponds to a graph, and color represents the evolutionary regime for the given fitness: blue color corresponds to the suppression regime and red color to amplification regime.
(EPS)

S6 Fig Graphs of order 8 with a double transition of type Suppressor/Amplifier/Suppressor. (EPS)
