# Supplementary material for: Evolutionary regime transitions in structured populations
Source: PLoS One. 2018 Nov 26;13(11):e0200670. doi: 10.1371/journal.pone.0200670 (PMC6261048; doi:10.1371/journal.pone.0200670)
Supplement: S1 Text — Proofs of some facts on the concavity of the function Φ0(r). (PDF) [file pone.0200670.s001.pdf]

S1 Text: On some properties of $\Phi_0(r)$ and its limit as $N \to \infty$

In this text we prove some facts about the rational function

$$\Phi_0(r) = \frac{r^{N-1}}{r^{N-1} + r^{N-2} + \cdots + 1}$$

stated in the main text of the paper.

$\Phi_0(r)$ uniformly converges to its limit

It is well known that the limit of $\Phi_0(r)$ as $N \to \infty$ is

$$\Phi_\infty(r) = \begin{cases} 0 & \text{if } r < 1 \\ 1 - \frac{1}{r} & \text{if } r \geq 1 \end{cases}$$

Result 1. *This convergence is uniform, in fact,*

$$\sup\{|\Phi_0(r) - \Phi_\infty(r)| \mid r \in (0, \infty)\} \leq \tfrac{1}{N}$$

Proof. A direct computation gives the result. For $r = 1$ there is nothing to prove. For $r < 1$,

$$|\Phi_0(r) - \Phi_\infty(r)| = \frac{r^{N-1}}{r^{N-1} + r^{N-2} + \cdots + 1}.$$

Since $r < 1$, $r^j < r^{j-1}$ for any $j \geq 0$. Therefore

$$1 + r + \cdots + r^{N-1} > r^{N-1} + \cdots + r^{N-1} = N\, r^{N-1},$$

So

$$\frac{r^{N-1}}{1 + r + \cdots + r^{N-1}} < \frac{1}{N}.$$

For $r > 1$

$$\begin{aligned}\Phi_0(r) - \Phi_\infty(r) &= \frac{r^{N-1}}{r^{N-1} + r^{N-2} + \cdots + 1} - 1 + \frac{1}{r} = \frac{-r^{N-2} - \cdots - 1}{r^{N-1} + r^{N-2} + \cdots + 1} + \frac{1}{r} \\ &= \frac{-r(r^{N-2} + \cdots + 1) + r^{N-1} + r^{N-2} + \cdots + 1}{r(r^{N-1} + r^{N-2} + \cdots + 1)} \\ &= \frac{1}{r(r^{N-1} + r^{N-2} + \cdots + 1)}\end{aligned}$$

Since $r > 1$, $r^j > 1$ for any $j \geq 1$, hence $r(r^{N-1} + r^{N-2} + \cdots + 1) > N$ and therefore $\Phi_0(r) - \Phi_\infty(r) < \frac{1}{N}$. □

First derivative convergence as $N \to \infty$

It is stated in the paper that the sequence of functions $\Phi_0(r)$ does not converge to $\Phi_\infty(r)$ in class C^1. It is obvious, since $\Phi_0(r)$ has continuous derivatives on $(0, \infty)$ while $\Phi_\infty(r)$ is not differentiable at $r = 1$.

It is interesting to see what happens to the limit of the derivatives at $r = 1$. A direct computation give us

$$\begin{aligned}\frac{\partial \Phi_0(r)}{\partial r}\bigg|_{r=1} &= \left[\frac{(N-1)r^{N-2}}{r^{N-1} + \cdots + 1} - \frac{r^{N-1}((N-1)r^{N-2} + \cdots + 2r + 1)}{(r^{N-1} + \cdots + 1)^2}\right]\Bigg|_{r=1} \\ &= \frac{N-1}{N} - \frac{\sum_{i=1}^{N-1} i}{N^2} = \frac{N-1}{2N} \xrightarrow[N\to\infty]{} \frac{1}{2}.\end{aligned} \tag{1}$$

On the other hand, while there is no derivative of $\Phi_\infty(r)$ at $r = 1$, it is possible to compute the slope of the *tangent from the right*. In other words, the derivative of $1 - \frac{1}{r}$ at $r = 1$, which is 1 (see Figure 1).

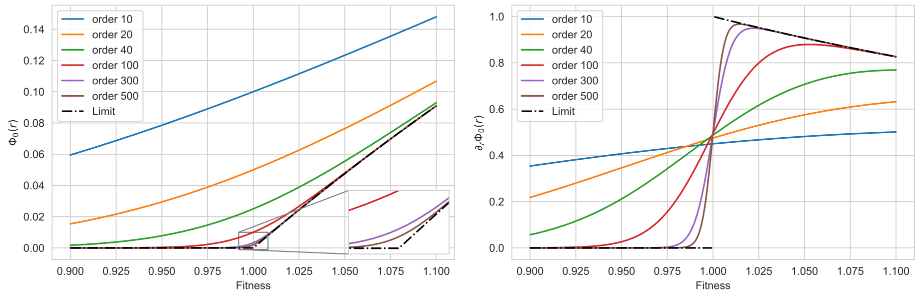

Figure 1: The convergence of $\Phi_0(r)$.

Concavity and convexity

The appearance of phase transitions in the sense of the paper is tied to the location of the inflection points of the functions $\Phi(r)$ of a given graph and $\Phi_0(r)$ of the complete graph of the same order. Exploring in full generality the location of those points in all graphs is impossible. But just the behavior of the simplest one, the homogeneous population, give us a grasp of the complexities hidden around $r = 1$.

Convexity and concavity can be computed from the sign of the second derivative. For small order, up to 5, it is possible to compute this derivative by hand to check that the function is in fact concave (see Figure 2).

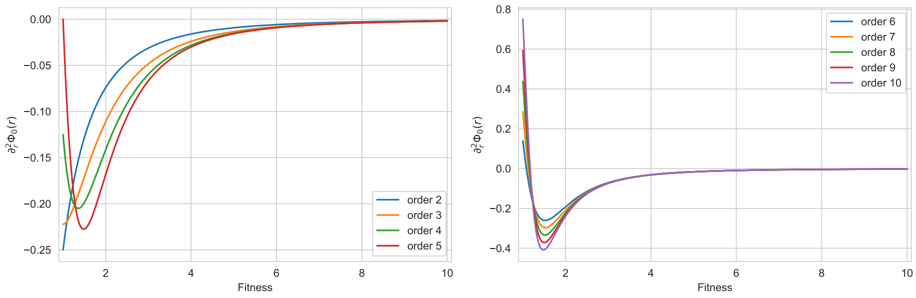

Figure 2: The second derivative of $\Phi_0(r)$ for small orders.

For greater orders the computation is just cumbersome, but it is possible to argue at some extent about it without the need of the direct computation.

The limit of $\Phi_0(r)$ as $r \to \infty$ is 1 and $\Phi_0(r) \neq 1$ for all $r > 0$, therefore, at least for large enough r's, $\Phi_0(r)$ should increase towards the asymptote 1, therefore, the second derivative should be negative for large enough r's. That is, the function is, for large values of its variable, concave.

But it is possible to compute the second derivative of the function for $r = 1$.

Denote $f(r) = \sum_{i=0}^{N-1} r^i$. From (1)

$$
\begin{aligned}
\frac{\partial^2 \Phi_0(r)}{\partial r^2} &= \frac{\partial}{\partial r}\left[\frac{(N-1)r^{N-2}}{f(r)} - \frac{r^{N-1}f'(r)}{(f(r))^2}\right] \\
&= \frac{(N-1)(N-2)r^{N-3}}{f(r)} - \frac{(N-1)r^{N-2}f'(r)}{f^2(r)} \\
&\quad - \frac{(N-1)r^{N-2}f'(r) + r^{N-1}f''(r)}{f^2(r)} + 2\frac{r^{N-1}(f'(r))^2}{f^3(r)} \\
&= \frac{(N-1)(N-2)r^{N-3}}{f(r)} - 2\frac{(N-1)r^{N-2}f'(r)}{f^2(r)} \\
&\quad - \frac{r^{N-1}f''(r)}{f^2(r)} + 2\frac{r^{N-1}(f'(r))^2}{f^3(r)}.
\end{aligned} \tag{2}
$$

It is obvious that $f(1) = N$, and $f'(1) = \sum_{i=1}^{N-1} i = \frac{1}{2}N(N-1)$. On the other hand, since $f''(r) = (N-1)(N-2)r^{N-3} + \cdots + 3 \cdot 2r + 2 \cdot 1$, we conclude that

$$
f''(1) = \sum_{i=1}^{N-2} i(i+1) = \frac{1}{3}N(N-1)(N-2).
$$

Substituting in (2)

$$
\begin{aligned}
\frac{\partial^2 \Phi_0(r)}{\partial r^2}\Big|_{r=1} &= \frac{(N-1)(N-2)}{N} - \frac{(N-1)^2}{N} - \frac{1}{3}\frac{(N-1)(N-2)}{N} + \frac{1}{2}\frac{(N-1)^2}{N} \\
&= \frac{\frac{2}{3}(N-1)(N-2)}{N} - \frac{\frac{1}{2}(N-1)^2}{N} \\
&= \frac{N^2 - 6N - 5}{6N}.
\end{aligned} \tag{3}
$$

The sign of (3) is given by the sign of $N^2 - 6N - 5$. This quantity is 0 for $N = 1$ and $N = 5$. Between those points is negative, and it is positive everywhere else.

Therefore,

Result 2. *For all orders greater or equal to* 6, $\Phi_0(r)$ *is convex around* 1, *so there is, at least, one inflection point in* $(1, \infty)$.
