# Supplementary material for: Evolutionary regime transitions in structured populations
Source: PLoS One. 2018 Nov 26;13(11):e0200670. doi: 10.1371/journal.pone.0200670 (PMC6261048; doi:10.1371/journal.pone.0200670)
Supplement: S1 Table — Number, place, and type of transitions for all connected graphs of order 7 or less. (PDF) [file pone.0200670.s004.pdf]

ID	Order	Number of cuts	Transition	Type
1	2			Isothermal
1027	3			Isothermal
526339	4			Isothermal
527367	4			Isothermal
135268355	5			Isothermal
135797775	5			Isothermal
1580044	5	1	1.78287498125135	Suppresor/Amplifier
17449355267	6			Isothermal
17450932235	6			Isothermal
17585679375	6			Isothermal
17586207775	6			Isothermal
17450408974	6			Isothermal
17586206722	6	1	1.22193837434221	Suppresor/Amplifier
17584109586	6	1	1.33095684176226	Suppresor/Amplifier
17586207760	6	1	1.36082690317112	Suppresor/Amplifier
406338584	6	1	1.87965125079520	Suppresor/Amplifier
17451461644	6	1	2.82789894107104	Suppresor/Amplifier
406331401	6	1	4.56890343185820	Suppresor/Amplifier
1134140852227	7			Isothermal
1134682454043	7			Isothermal
1151592840222	7			Isothermal
1151998655551	7			Isothermal
1151997603842	7	1	1.01291427227256	Suppresor/Amplifier
1151863361537	7	1	1.04010585578508	Suppresor/Amplifier
18127286304	7	1	1.11535825972254	Suppresor/Amplifier
1151997073410	7	1	1.11712985903186	Suppresor/Amplifier
1151591804932	7	1	1.13838192561540	Suppresor/Amplifier
34770286640	7	1	1.15320784414287	Suppresor/Amplifier
1151456532514	7	1	1.16112279449031	Suppresor/Amplifier
1151998654496	7	1	1.21650842766890	Suppresor/Amplifier
1151998101505	7	1	1.23782574145902	Suppresor/Amplifier
1151998114848	7	1	1.26569832076209	Suppresor/Amplifier
1151998115845	7	1	1.29194476891885	Suppresor/Amplifier
1117502071820	7	1	1.32558467744223	Suppresor/Amplifier
1151862332432	7	1	1.35798530459835	Suppresor/Amplifier
1151998640129	7	1	1.37227878517787	Suppresor/Amplifier
1134281915452	7	1	1.47472235317447	Suppresor/Amplifier
1151998655520	7	1	1.47753803217999	Suppresor/Amplifier
1151998648321	7	1	1.47874663370948	Suppresor/Amplifier
1151998654466	7	1	1.49561259905446	Suppresor/Amplifier
1151998129156	7	1	1.50428354562985	Suppresor/Amplifier
52487020545	7	1	1.52505989591938	Suppresor/Amplifier
1151990776850	7	1	1.68966928072836	Suppresor/Amplifier
1134818766881	7	1	1.77212949793012	Suppresor/Amplifier
52487027760	7	1	1.80462179670359	Suppresor/Amplifier
1151998640145	7	1	1.94308343834170	Suppresor/Amplifier
1151861813254	7	1	1.96627084630835	Suppresor/Amplifier
1100324302860	7	1	1.98160809559319	Suppresor/Amplifier
1151994460194	7	1	1.98527430207006	Suppresor/Amplifier
1134147168268	7	1	2.13899800717826	Suppresor/Amplifier
1151998648329	7	1	2.16540201237524	Suppresor/Amplifier
1151863909388	7	1	2.63107033615185	Suppresor/Amplifier
18127282217	7	1	2.85222067999417	Suppresor/Amplifier
1151992863778	7	1	2.89510857860393	Suppresor/Amplifier
1151461244933	7	1	2.95858912425240	Suppresor/Amplifier
1151058073628	7	1	2.96535184460917	Suppresor/Amplifier
1151998652455	7	1	3.18958946568448	Suppresor/Amplifier
1151993934886	7	1	3.38713149404028	Suppresor/Amplifier
52479149074	7	1	3.41989386804709	Suppresor/Amplifier
52487012369	7	1	3.51280495555811	Suppresor/Amplifier
1151996551171	7	1	3.93420590896262	Suppresor/Amplifier
1151996020755	7	1	4.06745679065459	Suppresor/Amplifier
1134682447881	7	1	4.77595089988740	Suppresor/Amplifier
1151592835082	7	1	4.98811715947310	Amplifier/Suppresor
1151864430605	7	1	5.08434009504437	Suppresor/Amplifier
1151998648333	7	1	5.17611822443887	Suppresor/Amplifier
1151860745228	7	1	6.37125003382654	Amplifier/Suppresor
1151994973210	7	1	6.86047703514633	Suppresor/Amplifier
1134549822492	7	1	6.88630847258735	Suppresor/Amplifier
1134416132124	7	1	8.93833010810699	Suppresor/Amplifier
1151864426517	7	1	10.45645176381780	Suppresor/Amplifier
1151997074462	7	1	13.61188814438320	Suppresor/Amplifier
1151592837126	7	1	24.79702019687890	Amplifier/Suppresor
1151998652423	7	1	25.47101313451720	Suppresor/Amplifier
1151590742058	7	1	77.28669194491090	Suppresor/Amplifier
1151997057027	7	1	79.15367807274810	Suppresor/Amplifier
1151592839194	7	1	85.75032790283100	Suppresor/Amplifier
