# Supplementary material for: Evolutionary regime transitions in structured populations
Source: PLoS One. 2018 Nov 26;13(11):e0200670. doi: 10.1371/journal.pone.0200670 (PMC6261048; doi:10.1371/journal.pone.0200670)
Supplement: S2 Table — (PDF) [file pone.0200670.s005.pdf]

Order	Number of cuts	Transition	Type
6	0		Suppresor
7	1	5.17611822443887	Suppresor/Amplifier
8	1	4.15078510378738	Suppresor/Amplifier
9	1	3.93544655943209	Suppresor/Amplifier
10	1	3.78058724025916	Suppresor/Amplifier
11	1	3.64258026377893	Suppresor/Amplifier
12	1	3.51997271830432	Suppresor/Amplifier
13	1	3.41211321933171	Suppresor/Amplifier
14	1	3.31714095639532	Suppresor/Amplifier
15	1	3.23289102702108	Suppresor/Amplifier
